# Climate risk assessment needs urgent improvement

**DOI:** 10.1038/s41467-022-31979-w

**Published:** 2022-08-25

**Authors:** Alberto Arribas, Ross Fairgrieve, Trevor Dhu, Juliet Bell, Rosalind Cornforth, Geoff Gooley, Chris J. Hilson, Amy Luers, Theodore G. Shepherd, Roger Street, Nick Wood

**Affiliations:** 1 Microsoft, Redmond, USA; 2 Walker Institute, University of Reading, Reading, UK; 3 CSIRO, Canberra, Australia; 4 Centre for Climate and Justice, University of Reading, Reading, UK; 5 Department of Meteorology, University of Reading, Reading, UK; 6 Environmental Change Institute, University of Oxford, Oxford, UK; 7 Climate Policy Research, Sydney, Australia

**Keywords:** Climate sciences, Climate change

## Abstract

Climate risk assessments are a key tool in planning for climate change impacts. This commentary examines the current underlying problems with them and puts forward a framework to provide solutions to improve their effectiveness.

## Teaser

Existing constraints in current climate risk assessments make them inappropriate to effectively assess the true exposure of society and businesses to climate-related risk. Using the key constraints to guide a conceptual framework, we identify four cross-cutting and inter-related critical paths for improvement.

The impacts of climate change are already posing a significant risk to biodiversity and human welfare^1^: every community and sector of the economy faces climate-related risks, including physical risk derived from climate variability and change, and transition risk derived from the social and economic transformations required to achieve a climate-resilient and net-zero future^2^.

The urgency and seriousness of this challenge are reflected in the rapidly emerging regulation for disclosure of climate-related risks across the world such as the recent proposal by US Securities and Exchange Commission and climate-related financial disclosure legislation in UK. Mandatory disclosure makes climate risk assessment (CRA) a critical matter not only for every organisation, but for their investors, lenders, and insurers.

Although the number of tools to support CRA has rapidly increased in the last few years, these tools have been found to suffer from major limitations^3,4^. CRA requires not only knowledge of the climate change hazards across multiple space and timescales (e.g., likelihood of changes to extreme rain over North America over the next decade), knowledge of the exposures (e.g., location of assets and value chains), and knowledge of the vulnerabilities (e.g., response of communities to drought or response of supply chain to changes in carbon taxes). Crucially, appropriate CRA also requires the ability to integrate all these heterogeneous sources of information—and their associated and unavoidable uncertainties—to evaluate the effectiveness of possible interventions, helping to communicate risk and prioritise investments. From this perspective of integration, we have identified three key constraints on the effectiveness of the current CRAs:

- ***Scope*** – today’s CRAs evaluate risks in isolation and do not fully consider compounding or systemic risks.
- ***Data*** – today’s CRAs typically use either top–down data that provide global coverage but are not locally robust, or bottom-up data that provide detailed local information but cannot be scaled globally.
- ***Transparency*** – today’s lack of commonly accepted methods and principles and the extensive use of ‘black-box’ approaches to CRA limits trust and the ability to improve, compare and combine the results of different assessments.

**Fig. 1:**
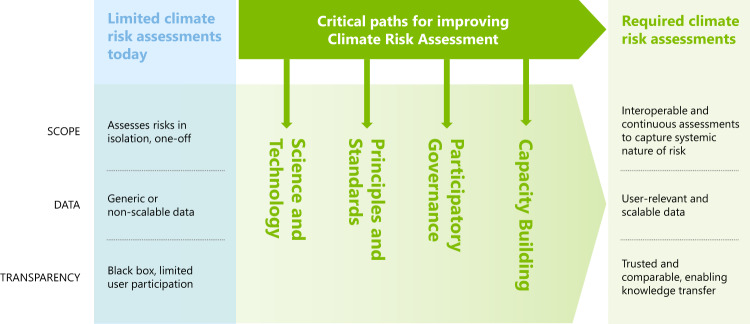
Critical paths for improving climate risk assessment. Diagram showing the key constraints (scope, data, transparency) making current climate risk assessment inappropriate to effectively assess climate-related risk, and the four identified cross-cutting and inter-related critical paths for improvement.

Using the existing key constraints as a guiding conceptual framework, and lessons learned from two sectors at the forefront of CRA—financial and humanitarian—we have identified four cross-cutting and inter-related critical paths for improvement—Science and Technology; Principles and Standards; Participatory Governance; and Capacity Building—that require urgent progress to enable the CRA needed by every organisation on the planet (Fig. 1).

## Current key constraints

### Scope

The scope of current approaches to CRA is typically too narrow, reflecting a focus on a single or a small group of issues. In addition, relevant stakeholders, be they a community or a business unit, are often not adequately represented in the definition of the scope. As a consequence, important context can be missed and multiple real-world risks that are inter-related, compounding and systemic are not considered, leading to a misestimation of the total climate risk^3^.

Commonly, individual corporate and humanitarian development CRAs tend to follow an ‘impact assessment’ approach: hazard-driven and focused on the impacts of climate change in a specific area or collection of assets. Such an assessment might, for example, consider the risk of drought to food production in a set of regions, or flood risks to datacentres worldwide. Albeit useful, this approach will miss compounding and systemic risks due to its limited scope. In the examples above, it would miss the risk to the agri-food industry from the combination of climate extremes with energy and geopolitical crises; and it would miss the risk for datacentre owners from climate policy changes in one region that could increase the cost of importing components from another, for example, the EU’s carbon border adjustment tax.

The financial sector has recently used ‘stress tests’—an approach to analyse how a particular system performs when subjected to extreme conditions—to assess the response of a sector, community or economic area to physical hazards and climate policy and economic decisions^5^. Although including both physical and transition risks, stress test approaches are still limited—only a few climate scenarios are considered and country or sector-wide factors are typically used—and often lack the granularity required to inform specific, immediate decisions by individual organisations and stakeholders. Financial institutions have been found to lack detailed information on the exact location and historical damage records of assets they hold as collateral^6,7^.

Moving forward, understanding the full breadth and depth of climate-related risks and enabling the assessment of compounding and systemic risks should be a priority. Realising this ambition and defining the appropriate scope of a CRA requires engagement and capacity building across the community of stakeholders through participatory governance.

Delivering the appropriate scope may need the integration of various individual risk assessments, each one with a limited scope. Emerging science and technology provide powerful capabilities which include scalability and rapid processing of information and uncertainties to enable such integration. For example, a financial institution assessing and reporting climate-related risks, as required by the Task Force on Climate-related Financial Disclosures, from investments in manufacturing may need to combine and integrate a CRA evaluating physical risks from flooding to manufacturing facilities, a CRA evaluating the transition risk from policy changes to the international manufacturing supply chain, and a CRA evaluating the transition risk from carbon taxes to customers in specific markets. Only by integrating all these individual risk assessments—ideally, dynamically and in real-time—would the financial institution and the manufacturer be able to understand its full risk exposure and the potential usefulness of its intended responses to the risk^8^. The effective integration of individual risk assessments requires standards for interoperability which, in turn, need principles and governance processes involving users, producers of CRA, data providers, and regulators.

### Data

Useful CRAs require a wide range of quantitative and qualitative data that are simultaneously relevant at local scales and consistent with the required scope globally. However, the necessary data are often difficult to access and use—e.g., climate data because of data volumes and domain-specific data formats. Data can also be complex and expensive to generate, such as exposure/vulnerability/policy datasets, and contain inherent uncertainties because of undetermined and unpredictable elements. In addition, as CRAs must be periodically updated, it can be challenging to acquire the necessary data because data providers may not update datasets with the required frequency or spatio-temporal resolution.

In the financial and corporate sector, these issues have typically been circumvented by using simplistic, top–down approaches that prioritise global coverage over local usefulness, like applying the same global climate dataset and downscaling approach everywhere regardless of differences in regional drivers of climate variability and change and user needs. Similarly, broad classifications of vulnerability and exposure are often applied globally, with the same damage function used for all office buildings irrespective of construction type or location. Such ‘cookie-cutter’ approaches to CRA are easy to scale and commoditise. However, this leads to information loss and hides critical local and time-horizon differences. In contrast, the humanitarian sector, where bottom-up assessments are common, often generates highly relevant local data such as prevailing sources of income and qualitative measures of adaptive capacity and livelihood resilience in a community^9^. Such local data is crucial to accurately assessing the resilience of systems—for example, linking information on crop vulnerability to information on people’s livelihoods—but the cost of collecting these data is high and the typical one-off nature of projects in the humanitarian sector often leads to organisational memory loss and processes that are not scalable^10^.

Moving forward, emerging science and technology provide useful ways to address current constraints around data. Cloud computing and machine learning have the potential to improve the tailoring of climate predictions at scale. Technologies such as drones, social media and mobile phones, combined with transparent data analytics, have the potential to reduce the cost and time required to create locally relevant and frequently updated geospatial and socio-economic datasets. However, accelerating the adoption of new capabilities and processes will require capacity building and knowledge generation and diffusion across stakeholders, in both the public and private sectors. In addition, local and system-based expert knowledge needs to be an intrinsic part of the end-to-end data architecture and governance. The reason is not only because of critical issues such as data equity and ethical concerns but also to ensure that assumptions and input information are valid and that outputs are targeted at assessing the climate resilience of social, economic and biodiversity systems^11,12^—making maladaptive practices less likely. This is required in every country, not only in developing countries^13^.

### Transparency

Transparency is critical to building trust in the conclusions of CRA; facilitating evaluation and comparison of assessments by communities, investors, regulators and other decision-makers; and enabling a continuous improvement feedback loop by sharing innovations and best practices. For CRAs to be useful, credible scientific methodologies, agreed taxonomies and definitions, and quality-controlled and reliable data must be employed.

However, the use of ‘black-box’, proprietary and unpublished methodologies is currently common, particularly in the financial sector. To a large extent, this is driven by a desire on the part of third-party service providers to commoditise the provision of CRAs for financial disclosure. This is a problem because it obscures the ability to evaluate and compare methodologies and it does not support knowledge diffusion. The lack of common principles, and the limited regulation of providers of such climate services, further compounds the problem^3^. In the humanitarian context, data and modelling approaches are generally open-source by default but the same lack of commonly accepted principles exists, making it difficult to compare assessments completed in different regions and to evaluate, integrate and scale up solutions^14^. In all sectors, the inherent and unavoidable uncertainty in climate risk demands full transparency in the assumptions, models and data used in the assessments. Particular care must be taken to avoid over-simplifications and “singular and definitive” statements that can lead to maladaptation^15^ when the reality is “plural and conditional”^16^.

Moving forward, emerging science and technology such as causal networks, which embed expert knowledge within Bayesian logic, can enable the transparent inclusion of heterogeneous data sources without sacrificing mathematical rigour^17^. Bayesian networks provide the ability to deal—transparently and including both quantitative and qualitative information that bridges the global and local scales consistently—with the unavoidable uncertainty in climate risk.

Additional common principles that can be adapted to enable transparent and robust assessments of climate risk include: ‘open secrets’ frameworks^18^ which have proven to be a useful way to share information between private and public organisations without foregoing the commercial value of such datasets; and open-access, peer-reviewed publications—provided that sufficient information on methods, metadata, data and software is made available—which can facilitate knowledge diffusion and local capacity building, contributing to the creation of organisational memory and increasing global capabilities to assess and manage climate risk.

## The way forward

Scope, data and transparency are three key constraints that make current CRAs inappropriate to effectively assess the true exposure of society and businesses to climate-related risk. The analysis of these constraints has enabled us to identify four interconnected critical paths for improvement (Fig. 1):

1. Expand the use of existing and emerging science and technology to enable better use of geospatial data and treatment of uncertainties.
2. Develop common principles and standards to enable transparency, comparison and interoperability of diverse and different risk assessments.
3. Implement participatory governance practices to enable the effective participation of multiple stakeholders.
4. Accelerate capacity building to enable innovation, knowledge generation and diffusion.

The potential of existing and emerging scientific and technological advances is unrealised across most CRAs, requiring an acceleration in the adoption of new observations such as in situ and remote sensing, and rapid and scalable data processing capabilities now provided by machine learning and cloud computing.

In addition, common principles and, where necessary due to technical or regulatory reasons, appropriate standards to enable comparison and interoperability of data and methodologies need to be developed. This is critical to enabling the integration of CRAs and realising the required scope. The purpose of common principles is not only to facilitate transparency but to ensure the inclusion of users’ needs, expertise and capabilities. Research bodies, regulators, and the private sector all have a role to play in the development of common principles. Existing initiatives like the World Climate Research Programme Lighthouse Activities, Task Force on Climate-related Financial Disclosures, and US Securities and Exchange Commission proposed rules for climate-related disclosures should be linked and bottom-up connections across multiple organisations and stakeholders should be facilitated and supported.

Governance practices that enable the participation of multiple stakeholders need to be implemented, and capacity building to enable knowledge generation and diffusion across stakeholders needs to be accelerated. Governance is critical to support appropriate scoping and to create the enabling environment required to deal with critical and difficult decisions around CRAs. The Regional Climate Consortium for Asia and the Pacific^19^ and the Climate Measurements Standard Initiative^20^ are good examples of a participatory approach with industry stakeholders to develop standardised scenario analysis for CRA for the financial and humanitarian sectors.

As international efforts to fight the COVID pandemic have demonstrated, trust and knowledge-sharing are crucial to enable the effective and rapid assessment required to manage systemic risks. Therefore, rapidly increasing the capacity and engagement of all stakeholders—ensuring the inclusion of those most vulnerable—to influence, implement and maintain end-to-end CRAs is not only an issue of climate justice but a critical component of increasing global resilience. Otherwise, the absence of adequate governance processes and knowledge asymmetries between providers and users can have negative unintended consequences such as improper “trading-out of climate risk”^21^.

Rapid progress in CRAs is eminently feasible. Our aim here is to facilitate the development of a common point of view across the risk assessment community to enable the joint pursuit of improvements along the four critical paths identified. Only by urgently improving CRAs will society and businesses be able to understand and manage climate-related risks before they lead to catastrophic, systemic failures.
